# Magnetic Fluid Deformable Mirror with a Two-Layer Layout of Actuators †

**DOI:** 10.3390/mi8030072

**Published:** 2017-03-02

**Authors:** Zhizheng Wu, Xianghui Kong, Zhu Zhang, Junqiu Wu, Tao Wang, Mei Liu

**Affiliations:** Department of Precision Mechanical Engineering, Shanghai University, Shanghai 200072, China; kongxianghuii@163.com (X.K.); zhuzhang2012@163.com (Z.Z.); wujunqiu_rachel@163.com (J.W.); wangt@shu.edu.cn (T.W.); mliu@shu.edu.cn (M.L.)

**Keywords:** magnetic fluid, deformable mirror, electromagnetic coil, two-layer, adaptive optics

## Abstract

In this paper, a new type of magnetic fluid deformable mirror (MFDM) with a two-layer layout of actuators is proposed to improve the correction performance for full-order aberrations with a high spatial resolution. The shape of the magnetic fluid surface is controlled by the combined magnetic field generated by the Maxwell coil and the two-layer array of miniature coils. The upper-layer actuators which have a small size and high density are used to compensate for small-amplitude high-order aberrations and the lower-layer actuators which have a big size and low density are used to correct large-amplitude low-order aberrations. The analytical model of this deformable mirror is established and the aberration correction performance is verified by the experimental results. As a new kind of wavefront corrector, the MFDM has major advantages such as large stroke, low cost, and easy scalability and fabrication.

## 1. Introduction

Adaptive optics (AO) is a technology that enables us to achieve complex aberration corrections for a wide range of applications [1,2]. Conventional AO systems utilize spatial light modulators [3,4] or solid deformable mirrors (DM) [5,6] to compensate for the phase fluctuations that result from non-uniformity in the properties of the medium through which light travels or imperfections in the geometry of the optical components. The spatial light modulators are available in both reflective as well as transparent modes. This type of wavefront corrector has the advantage of very high spatial resolution provided by extremely small liquid crystals. However, they are limited by the relatively small magnitude of correction that they can provide, usually in the range of a few micrometers. Solid deformable mirrors have evolved as the most widely used wavefront correction elements in optics systems, which can offer relatively high strokes. Generally, solid deformable mirrors consist of a solid reflecting membrane or plate surface to which an actuator structure is attached. Through manipulation of the actuators, the shape of the mirror can be modified to fulfill the compensation of distorted wavefronts. The common drawbacks of the solid deformable mirrors are the high cost per actuator channel and the complex fabrication process. Most of the currently available solid deformable mirrors offer small inter-actuator strokes and the maximum deflection magnitudes are limited to tens of micrometers.

In practice, studies have shown that in many applications such as laser beam shaping [7,8,9] and ophthalmic imaging systems [10,11,12], AO system needs to effectively deal with the low-amplitude high-order aberrations along with some low-order aberrations that come with very high amplitudes simultaneously. For instance, high-resolution retinal imaging technology based on AO plays an important role in vision science and will aid in the early clinical diagnosis of retinal diseases. In view of the characteristics of ocular aberrations for a large and diverse population, e.g., myopic eyes, a set of adaptive optics systems using two deformable mirrors have been designed [10,11,12]. The first large-stroke DM with a limited number of actuators is used to correct large-amplitude low-order aberrations, and the second one with a low stroke but a high spatial correction resolution is used to compensate for the small-amplitude high-order aberrations. However, its practical application in ophthalmology is restricted by the complexity and the high price.

In [13,14,15], a new type of liquid deformable mirror is proposed based on the actuation of the magnetic fluid. Though the disadvantage of the liquid mirror is that the mirror is constrained to remaining horizontal, the magnetic fluid deformable mirror (MFDM) has major advantages such as large strokes, low cost per actuator and easy scalability. The strokes of the single actuator or inter-actuator can both easily reach more than 100 μm with limited power consumption. However, in order to produce a large mirror surface deformation, the size of the electromagnetic coils is normally designed to be large, which results in a low density of actuators and thus is unfavorable for the correction of high-order aberrations. In order to realize the correction of full-order aberrations with high spatial resolution, the design of a two-layer layout of the miniature electromagnetic coils is adopted in this paper. The dynamics model of the mirror is established, and the aberration correction performance is verified by the simulation and experimental results. As a new kind of wavefront corrector, the proposed MFDM has major advantages such as large stroke, low cost, easy scalability and a simple fabrication process, and thus can be easily customized for different applications.

## 2. Design of Magnetic Fluid Deformable Mirror (MFDM)

**Figure 1:**
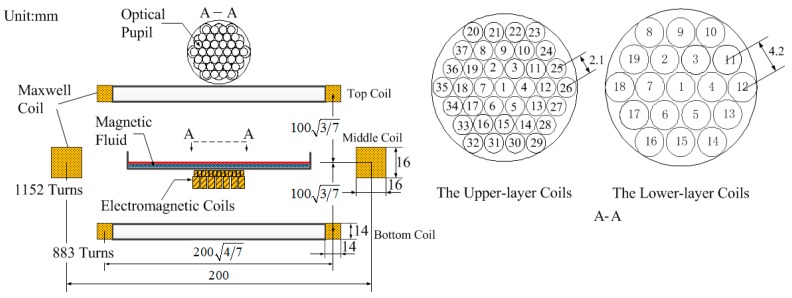
Schematic diagram of the prototype magnetic fluid deformable mirror (MFDM).

As shown in Figure 1, the primary elements of the MFDM are a layer of magnetic fluid, a thin film of a reflective material coated on the free surface of the fluid, a two-layer layout of the miniature electromagnetic coils placed underneath the fluid layer, and a Maxwell coil. The properties of the magnetic fluid used in this paper are given in Table 1, and they are stable colloidal suspensions of nano-sized, single-domain ferri/ferromagnetic particles and can be coated with a silver liquid-like thin film to improve the reflectance. 

In order to realize the correction of full-order aberrations with a high spatial resolution, the design of a two-layer layout of the miniature electromagnetic coils is adopted. As shown in Figure 1, the upper-layer actuators with a small size and high density are used to compensate for small-amplitude high-order aberrations and the lower-layer actuators with a big size and low density are used to correct large-amplitude low-order aberrations. The electromagnetic coils are conventional circular coils wound on a cylindrical bobbin, and the physical parameters are given in Table 2. Each layer of the coils is disposed in a hexagonal array. The upper-layer coils are radially spaced at 2.1 mm from center to center and the lower-layer coils are radially spaced at 4.2 mm, respectively. 

In order to linearize the response of the actuators, an external uniform magnetic field is produced by the Maxwell coil. As shown in Figure 1, the Maxwell coil consists of three separated coils, where each of the top and bottom coils should be of radius $\sqrt{\frac{4}{7}}R$, and distance $\sqrt{\frac{3}{7}}R$ from the plane of the middle coil of radius *R* = 100 mm [16]. The parameters are given in Table 3. The three separated coils wound with American wire gauge (AWG) 25 magnet wire follow the turn ratio of 64:49 for the top and bottom coil relative to the middle coil [16]. In addition, magnetic fluids typically show low reflectance to light and can be coated with silver liquid-like thin films to improve the reflectance [17,18]. In this paper, the self-assembly method is used to prepare the silver liquid-like thin film for the MFDM. Firstly, the solution of silver nano-particles was dissociated by centrifugation to remove the supernatant, and ethanol was then infused to purify the silver nano-particles. The obtained silver nano-particles were added into the mixed solution of ethanol and dodecanethiol, and then centrifuged after being kept at room temperature for 24 h. Finally, the ethyl acetate was added to the silver nano-particles obtained from the above step, and this solution was then added drop by drop to the surface of the magnetic fluid. After the ethyl acetate evaporated, the hydrophobic dodecanethiol encapsulated silver nano-particles automatically stacked and spread on the surface of the magnetic fluid to form a large-scale domain of silver liquid-like film.

**Figure 2:**
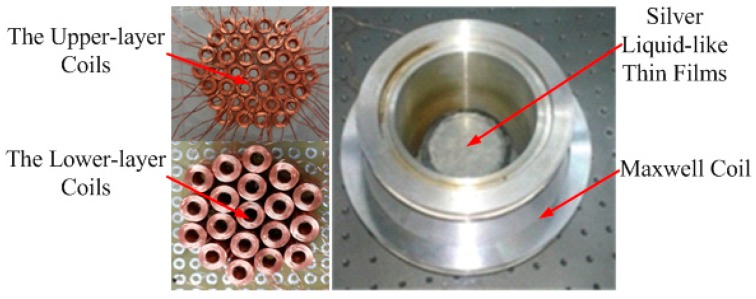
Assembly of the prototype MFDM.

A snapshot of the assembly of the mirror is shown in Figure 2. The two-layer layout of the miniature electromagnetic coils is placed within the Maxwell coil and a container filled with a 1-mm-deep layer of ferrofluid sits on top of the miniature coils, which are coated with the thin silver liquid-like film.

## 3. Analytical Surface Dynamics Model of MFDM

**Figure 3:**
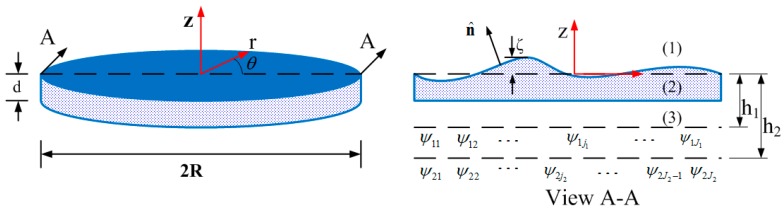
Geometric representation of a circular MFDM.

The proposed MFDM is represented by a cylindrical horizontal layer of a magnetic fluid as shown in Figure 3. The top free surface of the fluid layer is coated with a reflective film and serves as the deformable surface of the mirror. The deflection of the mirror surface at point (*r_k_*, θ*_k_*) is denoted by ζ(*r_k_*, θ*_k_*, *t*), where *k* = 1,2,3,…,*K* is a discrete number of surface locations. The magnetic field generated by any given coil, centered at the horizontal location (*r_ij_*, θ*_ij_*), is idealized as that of a point source of magnetic potential ψ*_ij_*(*t*), where *i* = 1,2 is the *i*th layer of actuators, and *j*, *j* = 1,2,3,…, *J_i_* is the *j*th coil of each layer.

The magnetic field itself is governed by Maxwell’s equations. Since the magnetic field of the miniature coils is idealized as that of point sources of magnetic potential located at the fluid domain boundary, a current-free electromagnetic field can be assumed. Using this assumption and further assuming that the displacement currents in the fluid are negligible, Maxwell’s equations can be written as:

(1) ∇×H=0 , ∇⋅B=0

where **B** is the magnetic flux density, which is related to the magnetic field **H** and the magnetization **M** by the following constitutive relationship:

(2) $$B=\mu H=\mu_{0}(H+M)$$

where μ is the magnetic permeability of the magnetic fluid, μ_0_ is the magnetic permeability of free space. Assuming the magnetic fluid is linearly magnetized by the applied field, the magnetization vector **M** can be written as

(3) M=χH

where $\chi =\frac{\mu }{\mu_{0}}-1$ is considered to be a constant. Considering that the magnetic field extends into the space above and below the fluid layer, Maxwell’s equations are applied to all three sub-domains marked in Figure 3 as (1)–(3). The scalar potentials ψ^(*l*)^, *l* = 1,2,3 describe the magnetic field vectors **H**^(*l*)^ in these sub-domains as follows:

(4) $$H^{\left(l\right)}=-\nabla \psi^{\left(l\right)},\;l=1,2,3$$

Using Equations (2)–(4), the magnetic flux density **B**^(*l*)^ in these sub-domains can be written in terms of the scalar potentials ψ^(*l*)^, *l* = 1,2,3 as

(5) $$B^{\left(l\right)}=-\mu_{0}(1+\chi )\nabla \psi^{\left(l\right)},\;l=1,2,3$$

The magnetic flux density **B** meets the principle of superposition. Assume the fluid is irrotational, then based on the principles of conservation of mass and momentum and the theories of magnetic fields, the perturbation part of the surface dynamic governing equations can be written as [19]:

(6) $$\nabla^{2}\phi =0,\;-d\leq z\leq \zeta$$

(7) $$\nabla^{2}\psi^{\left(l\right)}=0,\;l=1,2,3$$

(8) $$-\rho \frac{\partial \phi }{\partial t}+\rho g\zeta +\chi B_{0}\frac{\partial \psi^{\left(2\right)}}{\partial z}-\sigma (\frac{\partial^{\left(2\right)}\zeta }{\partial r^{\left(2\right)}}+\frac{1}{r}\frac{\partial \zeta }{\partial r}+\frac{1}{r^{\left(2\right)}}\frac{\partial^{\left(2\right)}\zeta }{\partial \theta^{\left(2\right)}})=0,\;z=\zeta$$

where ρ is the density of the fluid, σ is the surface tension, φ and ψ^(*l*)^, *l* = 1,2,3 are the perturbation components of the fluid velocity potential and the magnetic potential, respectively. Using the following two boundary conditions:

$$-\frac{\partial \phi }{\partial z}=\frac{\partial \zeta }{\partial t},\;z=\zeta$$

$$-\frac{\partial \phi }{\partial z}=0,\;z=-d$$

The solutions with respect to the input ψ*_ij_*(*t*) thus are obtained as follows:

(9) $$\zeta (r_{k},\theta_{k},t)=\;{\overset{\text{\textasciitilde{}}}{\zeta }}_{ij}(t)J_{m}(\lambda r_{k})\Theta (\theta_{k})$$

(10) $$\phi (r_{k},\theta_{k},z,t)=-\frac{1}{\lambda }\frac{\cosh[\lambda (z+d)]}{\sinh(\lambda d)}\frac{d\;{\overset{\text{\textasciitilde{}}}{\zeta }}_{ij}}{dt}J_{m}(\lambda r_{k})\Theta (\theta_{k})$$

(11) $$\psi^{\left(2\right)}(r_{k},\theta_{k},z,t)=-[A_{ij}(t)(\frac{\mu }{\mu_{0}}\cosh(\lambda z)-\sinh(\lambda z))+\frac{\chi }{\mu }B_{0}\;{\overset{\text{\textasciitilde{}}}{\zeta }}_{ij}(t)\cosh(\lambda z)]J_{m}(\lambda r_{k})\Theta (\theta_{k})$$

where $J_{m}(\cdot )$ is the Bessel function of the first kind, λ is the separation constant, and

$$\begin{array}{c} \Theta (\theta )=\left\{ \begin{array}{l} \sin\;m\theta ,\;m=1,2,3\cdots \\ \cos\;m\theta ,\;m=0,1,2,3\cdots \end{array}\right.\\ A_{ij}(t)=\frac{1}{Y(-\lambda h_{i})}\times \{Z(-\lambda h_{i})B_{0}\;{\overset{\text{\textasciitilde{}}}{\zeta }}_{ij}(t)+\frac{\kappa }{\pi R^{2}{\left[J_{m+1}(\varepsilon )\right]}^{2}}\psi_{ij}(t)R(r_{ij})\Theta (\theta_{ij})\}\\ Y(-\lambda h_{i})=-\frac{1}{\tanh(\lambda d)-\coth(\lambda d)}\times \{(\frac{\mu }{\mu_{0}}\alpha +\chi )\cosh(-\lambda h_{i})-[\frac{\mu }{\mu_{0}}(\alpha -\chi )-\frac{\chi^{2}}{\alpha }]\sinh(-\lambda h_{i})\}\\ Z(-\lambda h_{i})=\frac{1}{\tanh(\lambda d)-\coth(\lambda d)}\times [\alpha \cosh(-\lambda h_{i})+\chi \;\sinh(-\lambda h_{i})]\frac{\chi }{\mu }\\ \alpha =\frac{\mu }{\mu_{0}}\tanh(\lambda d)-\coth(\lambda d),\;\kappa =\{\begin{array}{c} 1\;m=0\\ 2\;m\neq 0 \end{array} \end{array}$$

Considering that the miniature coils are located far from the walls of the fluid container, so at *r* = *R* yields *J_m_*(λ*R*) = 0, which can be solved numerically and yields an infinite number of solutions ε*_mn_* = λ*R*, *m* = 0,1,2,…, *n* = 1,2,3,…, providing the eigenvalue λ*_mn_* for each mode as λ*_mn_* = ε*_mn_*/*R*. Combining *J_m_*(λ*r*) and Θ(θ), we define the following mode shapes as *H_mnc_* = *J_m_*(λ*_mn_r*)cos*m*θ and *H_mns_* = *J_m_*(λ*_mn_r*)sin*m*θ. 

For any coil ψ*_ij_*(*t*) on each layer, based on Equation (8) and the damping effect associated with the fluid viscosity η, the following surface dynamic equation with respect to the mode shape *H_mnc_* can then be obtained as:

(12) $$\frac{d^{2}\;{\overset{\text{\textasciitilde{}}}{\zeta }}_{ijmnc}(t)}{dt^{2}}+4\frac{\eta }{\rho }\lambda_{mn}^{2}\frac{d\;{\overset{\text{\textasciitilde{}}}{\zeta }}_{ijmnc}(t)}{dt}+\omega_{imn}^{2}\;{\overset{\text{\textasciitilde{}}}{\zeta }}_{ijmnc}(t)=-\frac{\chi }{\rho }B_{0}\frac{\tanh(\lambda_{mn}d)}{Y(-\lambda_{mn}h_{i})}\lambda_{mn}^{2}\frac{k}{\pi R^{2}{\left[J_{m+1}(\varepsilon_{mn})\right]}^{2}}\psi_{ij}(t)H_{mnc}(r_{ij},\theta_{ij})$$

where

$$\omega_{imn}^{2}=g\;\tanh(\lambda_{mn}d)\lambda_{mn}+\frac{\sigma }{\rho }\tanh(\lambda_{mn}d)\lambda_{mn}^{3}+\frac{\chi }{\rho }B_{0}^{2}\tanh(\lambda_{mn}d)\lambda_{mn}^{2}\frac{Z(-\lambda_{mn}h_{i})}{Y(-\lambda_{mn}h_{i})}$$

*m* = 0,1,2,… and *n* = 1,2,3,…

The main idea of derivation of Equation (12) is similar to the result of MFDM with a single-layer layout of actuators and more details can be found in [19]. A similar set of equations can be obtained with respect to the mode shape *H_mns_* as:

(13) $$\frac{d^{2}\;{\overset{\text{\textasciitilde{}}}{\zeta }}_{ijmns}(t)}{dt^{2}}+4\frac{\eta }{\rho }\lambda_{mn}^{2}\frac{d\;{\overset{\text{\textasciitilde{}}}{\zeta }}_{ijmns}(t)}{dt}+\omega_{imn}^{2}\;{\overset{\text{\textasciitilde{}}}{\zeta }}_{ijmns}(t)=-\frac{\chi }{\rho }B_{0}\frac{\tanh(\lambda_{mn}d)}{Y(-\lambda_{mn}h_{i})}\lambda_{mn}^{2}\frac{k}{\pi R^{2}{\left[J_{m+1}(\varepsilon_{mn})\right]}^{2}}\psi_{ij}(t)H_{mns}(r_{ij},\theta_{ij})$$

where *m* and *n* = 1,2,3,…

The generalized displacements ${\overset{\text{\textasciitilde{}}}{\zeta }}_{ijmnc}(t)$ and ${\overset{\text{\textasciitilde{}}}{\zeta }}_{ijmns}(t)$, obtained from the solution of the second-order differential Equations (12) and (13) respectively, and the corresponding mode shapes *H_mnc_* and *H_mns_* evaluated at any desired location (*r_k_*, θ*_k_*), give the total surface displacement at the location as

(14) $$\zeta (r_{k},\theta_{k},t)=\sum_{i=1}^{2}\sum_{j=1}^{J_{i}}\sum_{m=0}^{\infty }\sum_{n=1}^{\infty }\;{\overset{\text{\textasciitilde{}}}{\zeta }}_{ijmnc}(t)H_{mnc}(r_{k},\theta_{k})+\sum_{i=1}^{2}\sum_{j=1}^{J_{i}}\sum_{m=1}^{\infty }\sum_{n=1}^{\infty }\;{\overset{\text{\textasciitilde{}}}{\zeta }}_{ijmns}(t)H_{mns}(r_{k},\theta_{k})$$

Based on Equations (12)–(14), it can be seen that the surface response ζ(*r_k_*, θ*_k_*, *t*) is linearly dependent on the input ψ*_ij_*(*t*) introduced by each electromagnetic coil.

It should be noted that using Equations (12)–(14), the static surface response model of the mirror with respect to the perturbed magnetic field produced by each actuator can be obtained. Then the parameters of the coils in both layers as listed in Table 2 are designed based on the static model of MFDM so that the desired surface deflection of 5 µm by the single actuator in the upper layer and the deflection of 40 µm by the one in lower layer can be both produced. The ratio of the diameters of the coils in the lower and upper layers is finally rounded to have a factor of two so that the same pupil can be covered by the actuators in each layer.

## 4. Static Simulation of MFDM

**Figure 4:**
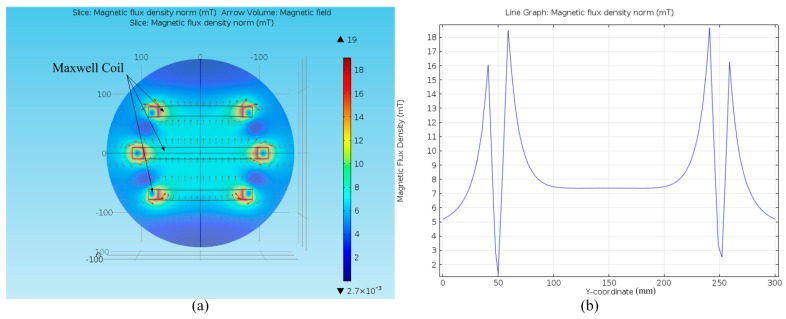
(**a**) The geometric model of Maxwell coil in COMSOL; (**b**) the curve of the magnetic field distribution on the center plane.

**Figure 5:**
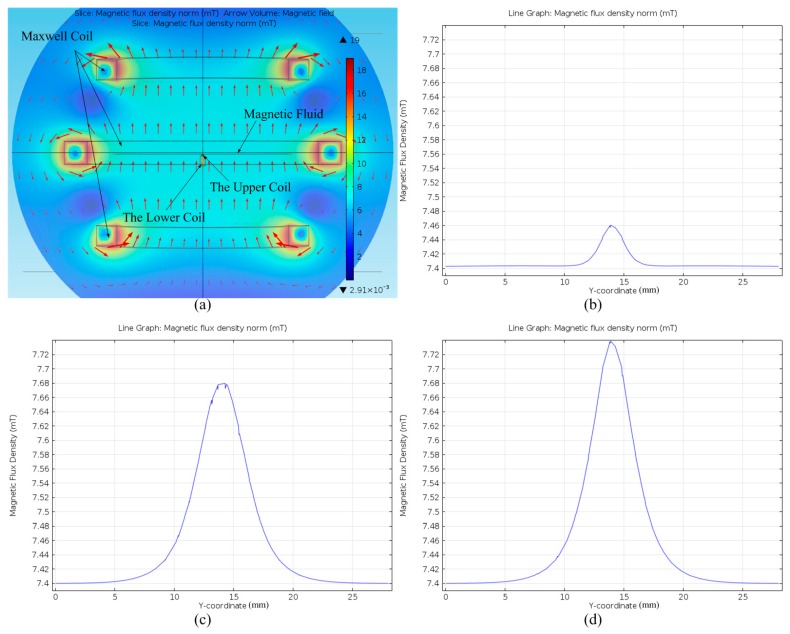
(**a**) The geometric model of MFDM in COMSOL; (**b**) the superposition of magnetic fields of the center coil in the upper layer and the Maxwell coil on the mirror surface; (**c**) the superposition of magnetic fields of the center coil in the lower layer and Maxwell coil on the mirror surface; (**d**) the superposition of magnetic fields of the two center coils and Maxwell coil on the mirror surface.

Based on the parameters listed in Table 1, Table 2 and Table 3, the magnetic fields of the Maxwell coil and the two layer coils are simulated using COMSOL Multiphysics (version 4.4, COMSOL Inc., Stockholm, Sweden). As shown in Figure 4a, the magnetic field inside the Maxwell coil is uniformly distributed. When the input current of the Maxwell coil is 500 mA, the uniform magnetic field intensity at the center plane can reach 7.4 mT (see in Figure 4b). Figure 5a shows the geometric model of the Maxwell coil, the center coils in the upper layer and lower layer and the magnetic fluid in COMSOL. Figure 5b,c show the superposition of the magnetic field distribution curve generated by the two center coils along with the Maxwell coil on the mirror surface, respectively. It is indicated that the maximum perturbed magnetic field intensity produced by the coil in the upper layer with a current of 35 mA can reach 0.06 mT on the mirror surface, whereas the maximum perturbed magnetic field intensity produced by the coil in the lower layer with a current of 50 mA can reach up to 0.28 mT. Based on the dynamics model of Equation (14), it can be derived that the maximum mirror surface displacements driven by the center coil in the upper layer or lower layer can reach up to 5 µm and 40 µm, respectively. Figure 5d shows the superposition result of the magnetic field driven by the two center coils and the Maxwell coil together, and the maximum magnetic field intensity on the mirror surface reaches 7.74 mT.

**Figure 6:**
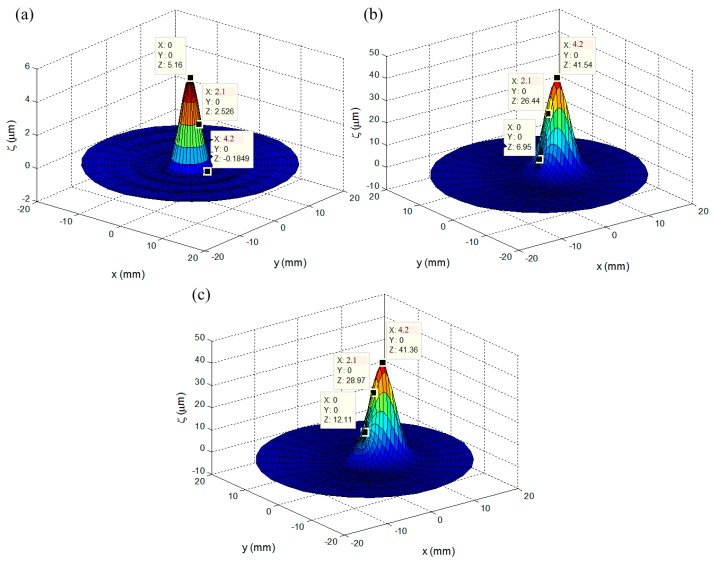
The surface deflection contour of the MFDM; (**a**) coil 1 in the upper layer is active; (**b**) coil 2 in the lower layer is active; (**c**) both coils are active. The measured points are selected at (0,0), (2.1,0), (4.2,0), respectively.

The linear response of the mirror surface deflection is simulated and verified in the COMSOL and MATLAB (version 2011a, Mathworks Inc., Natick, MA, USA) co-simulation environment. The lower-layer coils can provide a large stroke deflection due to the relatively large size of the electromagnetic coils compared with the upper-layer coils. Based on the structure parameters of the coils in each layer, the corresponding magnetic potentials are obtained in COMSOL with the analytical model of the MFDM developed in MATLAB. When the Maxwell coil is turned on with a current of 500 mA, the simulation results of the surface deflection contour are shown in Figure 6. In Figure 6a, it can be seen that the maximum deflection magnitude is 5.16 µm at (0,0) when coil 1 in the upper layer (see Figure 1) is active at 35 mA. In Figure 6b, the maximum deflection magnitude is 41.54 µm at (4.2,0) when coil 2 in the lower layer (see Figure 1) is set to 50 mA. When both coils are active, Figure 6c indicates that the surface deflection of the mirror is the linear sum of the deflections generated by each of the two coils separately.

## 5. Experimental Results

### 5.1. Linear Additivity of the MFDM Response

**Figure 7:**
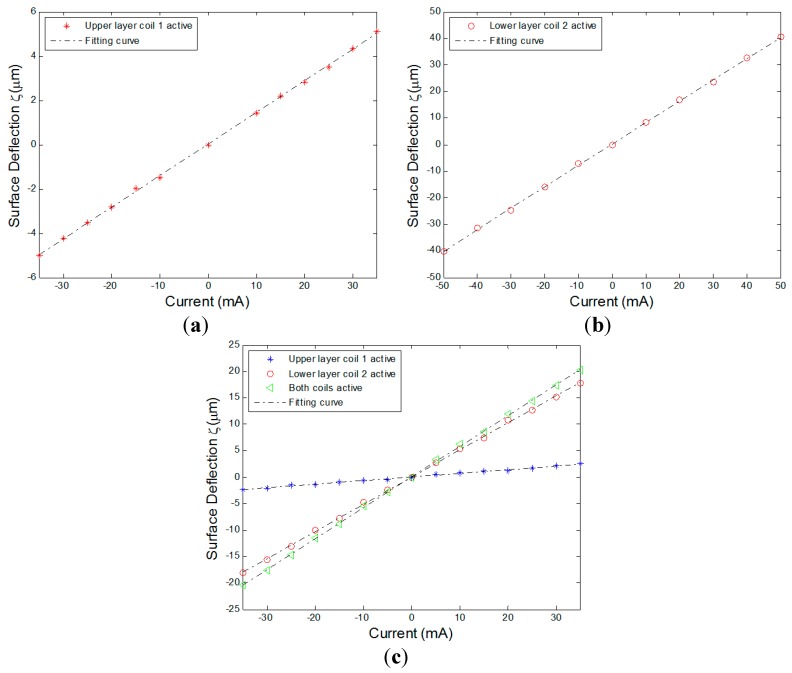
The experimental response of the MFDM; (**a**) coil 1 in the upper layer is active; (**b**) coil 2 in the lower layer is active; (**c**) both coils are active.

**Figure 8:**
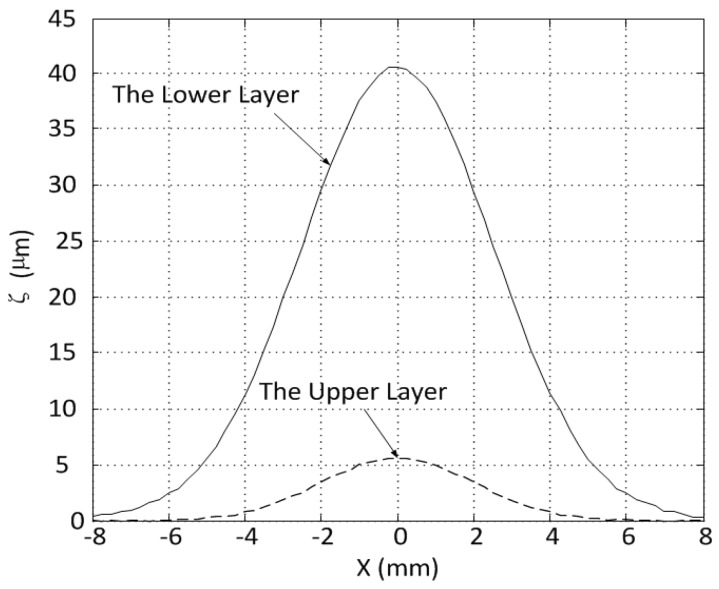
The influence functions of a single coil in the upper layer and lower layer of the MFDM.

In this section, the experimental results of the surface response of the mirror for different cases are presented to verify the response characteristics of the MFDM. The surface deflection with respect to the different input currents was measured using Polytec OFV 5000/552 and VIB-A-T31. During operation, the Maxwell coil was driven with a constant current of 500 mA, which produced a measured 7.43 mT uniform magnetic field inside of the Maxwell coil. In Figure 7a, the points marked as “*” signify the peak surface deflections of the MFDM when coil 1 in the upper layer (see Figure 1) is active. The experimental peak surface deflections for the case when coil 2 in the lower layer (see Figure 1) is active are marked as “ο”, as shown in Figure 7b. It is obvious that the surface deflections varied linearly with the increasing currents and both negative and positive deflections were achieved. As illustrated in Figure 7c, when the two coils are energized, the surface deflection (“Δ”) at a point midway between the two coils is the linear sum of the deflections (“*” and “ο”) at the point generated by each of the two coils separately. Similar to the simulation results, the maximum surface deflections driven by the single upper-layer or lower-layer coil can reach up to 5 µm and 40 µm, respectively. The corresponding influence functions of a single coil in the upper layer with an applied current of 35 mA or the one in the lower layer with a current of 50 mA are shown in Figure 8. As can be observed from the figure, the application of current to a single coil resulted in a Gaussian surface shape with its peak located immediately above the location of the energized coil, corresponding to a neighbor coupling constant of about 49% and 17% for the upper-layer and lower-layer actuators, respectively.

### 5.2. Dynamical Response

**Figure 9:**
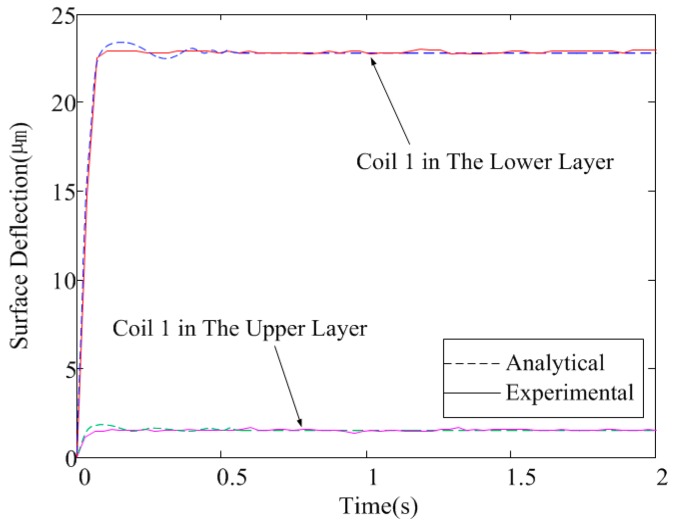
Dynamic response of the MFDM to a step input.

**Figure 10:**
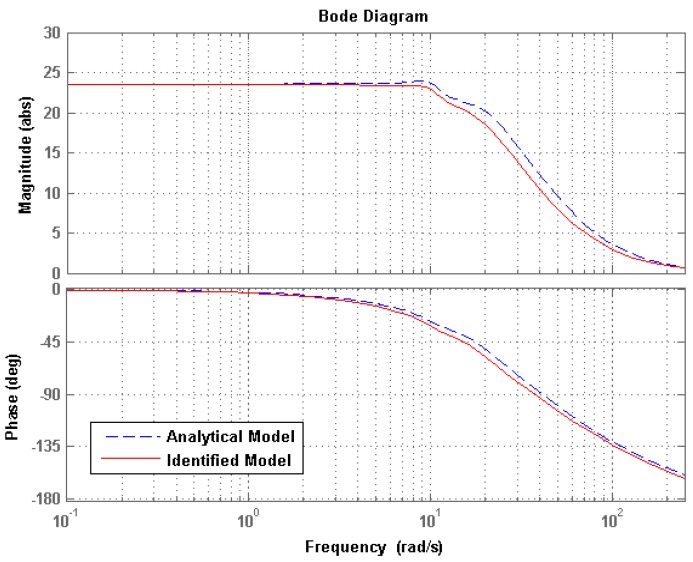
Bode plot of the MFDM system.

Figure 9 presents the dynamic response of the MFDM surface to a step input current signal, where the step currents of 10 and 25 mA are applied to the center coils in the upper layer and lower layer (coil 1 in Figure 1) of the MFDM, respectively. The time history of the mirror surface deflections above the center of the coils corresponding to these two inputs were noted and plotted. The response of the actual mirror surface is shown by the solid line while the analytically determined surface deflections are represented by the dashed line. As can be observed from the figure, the response given by the analytical model agreed well with the experimental results. The dynamics properties of the MFDM were also evaluated with a sweeping input current signal whose frequency varied from 0 to 50 Hz over time. Figure 10 shows the Bode plots of the experimentally identified model of the MFDM along with the analytical model, where the input current of 25 mA was applied at the center coil in the lower layer of the MFDM. The MFDM features a bandwidth of approximately 3 Hz. The dynamic bandwidth of MFDM can be improved by properly choosing the physical parameters of magnetic fluid. For example, by increasing the viscosity of the magnetic fluid along with an overdrive technique, experiments have shown a bandwidth of 1 kHz for the MFDM with a viscosity of 494 cP in [14].

### 5.3. Tracking of a Conical Surface Shape

**Figure 11:**
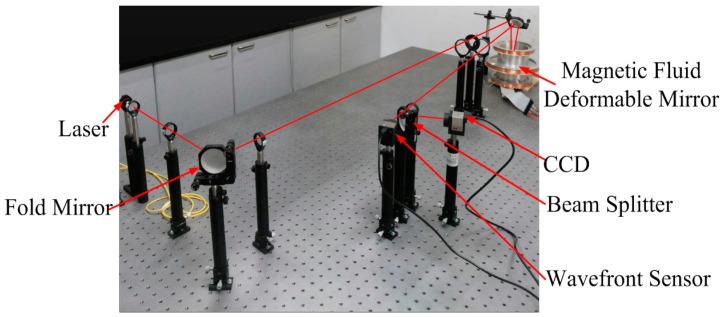
Snapshot of the experimental setup.

Based on the fabricated prototype of the MFDM, an experimental AO system was set up to evaluate the performance of the deformable mirror. The experimental arrangement is illustrated in Figure 11. A collimated, aberration-free beam of light from the laser source was magnified (×2.5) using the first optical relay. Then the beam was diverted and further magnified (×6) using the second relay. The magnified beam was limited by an aperture stop with the diameter of 20 mm. The 20 mm beam was directed on the horizontal fluid surface using the tip-tilt mirror, which also collected the reflected beam and folded it back to the wavefront sensor from the incoming beam. The reflected beam was de-magnified (×6.67) in order to be projected fully on the lenslet arrays of the wavefront sensor and the charge coupled device (CCD) The wavefront slope data were measured discretely at the 31 × 31 subapertures of the wavefront sensor and then transfered into the computer system. In the experimental evaluation, the mirror was supposed to produce a desired conical surface shape that was set to emulate an axicon.

**Figure 12:**
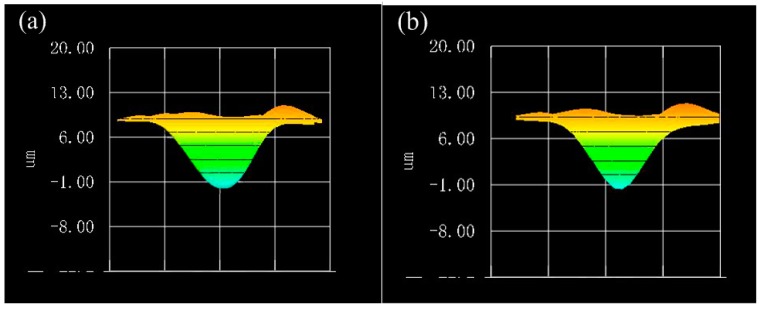
Comparison of conical surface shape produced by the MFDM with the actuators of the lower layer (**a**) versus the actuators of both layers (**b**).

Figure 12 shows the three-dimensional conical surface produced by the MFDM and recorded by the wavefront sensor. The conical surface shape as shown in Figure 12a was produced by the MFDM only with the lower-layer layout of actuators, and the resulting average root mean square (RMS) error was 0.4657 μm. In Figure 12b, the conical surface shape was produced by the MFDM with the two-layer layout of actuators. Due to the correction of the upper-layer coils, a more accurate conical surface shape was obtained and the average RMS error was decreased to 0.1920 μm.

### 5.4. Correction of Aberrations

**Figure 13:**
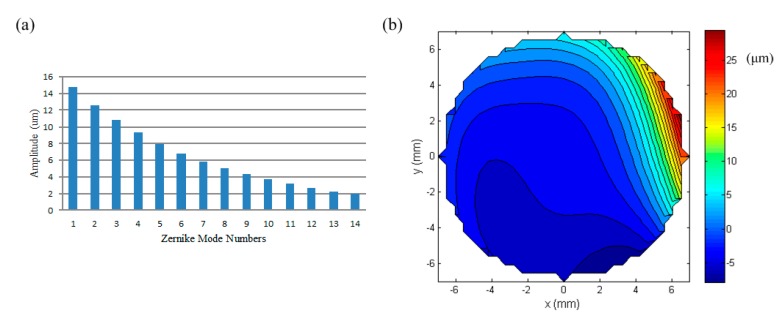
Typical aberration in Zernike modes; (**a**) Zernike mode numbers; (**b**) the target wavefront aberration.

**Figure 14:**
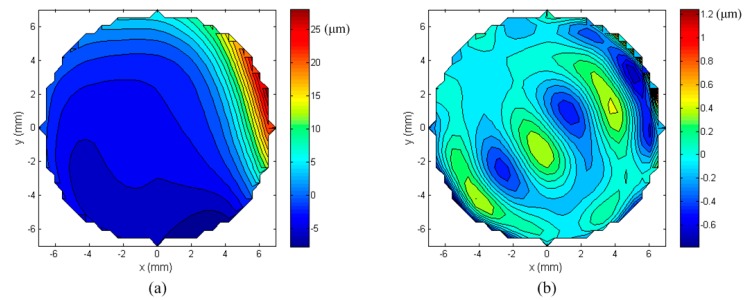
(**a**) Wavefront obtained by the proposed MFDM; (**b**) the residual wavefront error.

In this section, the mirror was used to produce a targeted wavefront which was expressed as a combination of typical Zernike modes. The amplitudes of each Zernike mode, Z_1_ to Z_14_, is shown in Figure 13a and the corresponding wavefront with a RMS value of 5.6341 μm is shown in Figure 13b. The input currents of the lower-layer coils of the MFDM were first calculated on account of the aberration with all 14 Zernike modes, and then the upper-layer coils were activated to correct the resulting residual wavefront error produced by the lower-layer coils. The final produced wavefront is presented in Figure 14, which shows a residual wavefront RMS error of 0.2263 μm.

**Figure 15:**
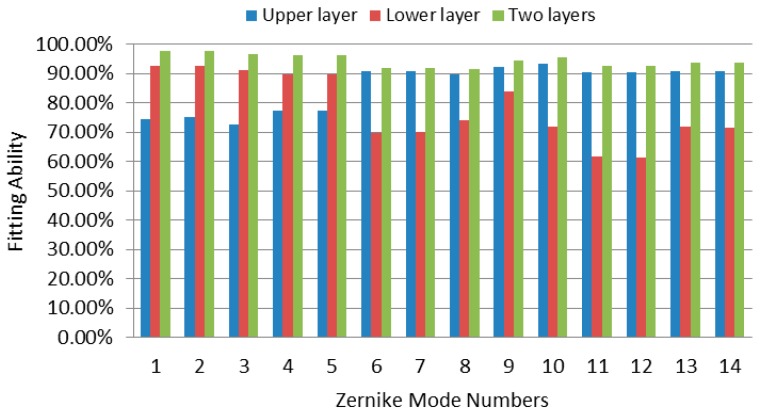
The aberration correction performance of the MFDM with respect to different Zernike modes.

Figure 15 shows the effectiveness of the MFDM for the correction of aberrations in each Zernike mode, as shown in Figure 13a. The *Y* axis displays the fitting ability of the MFDM for each Zernike mode, which is calculated as $\left(1-\sqrt{\sum_{i=1}^{961}{\left({\tilde{w}}_{i}-w_{i}\right)}^{2}\;}/\sqrt{\sum_{i=1}^{961}w_{i}^{2}}\right)$, where ${\tilde{w}}_{i}$ and $w_{i}$ denote the measured and the target wavefront values at the 31 × 31 subaperture positions of the wavefront sensor, respectively. The blue bars in Figure 15 show the correction capability of the upper-layer coils for different Zernike modes. It indicates that the upper-layer coils have a high correction performance for high-order aberrations, but the performance decreases for low-order aberrations, mainly due to the small amplitude of the surface deformation they can produce. However, the correction capability of the lower-layer coils is contrary. The red bars in Figure 15 indicate their high correction capability for low-order aberrations, but the correction performance drops down for high-order aberrations due to the low density of the lower-layer actuators. If both layers of the coils are activated, as seen from the green bars in Figure 15, the correction capability of the MFDM can be improved for all Zernike modes. These comparison results illustrate that the proposed MFDM with a two-layer layout of actuators can effectively improve the correction performance, especially for the cases that deal with aberrations featuring high-amplitude low-order modes and low-amplitude high-order modes simultaneously.

## 6. Conclusions

In order to improve the correction performance of the MFDM for full-order aberrations, a new MFDM with a two-layer layout of actuators is proposed in this paper. The structure and designed parameters of the MFDM were first presented. Then the dynamics model of the mirror was established and the corresponding mirror surface deformation performance was simulated using the COMSOL and MATLAB software packages. Finally, based on the fabricated prototype of the MFDM, an experimental AO system was set up to further evaluate the correction performance of the deformable mirror and the experimental results illustrated the effectiveness of the proposed MFDM to correct full-order aberrations for adaptive optics systems. 

## Figures and Tables

**Table 1:** Parameters of the magnetic fluid.

Magnetic Fluid	Parameters
Saturation magnetization	22 mT
Relative permeability	2.89
Density	1190 kg/m^3^
Viscosity	3 cP
Thickness	1 mm

**Table 2:** Parameters of miniature electromagnetic coil. AWG: American wire gauge.

Electromagnetic Coil	Parameters	
Position	Upper	Lower
Core-type	Air-cored	Air-cored
Material	Copper	Copper
Wire gauge	AWG37	AWG36
Internal diameter	1 mm	2 mm
External diameter	2 mm	4 mm
Length	1 mm	8 mm

**Table 3:** Parameters of the Maxwell coil.

Maxwell Coil	Parameters
Nominal diameter of the middle coil	200 mm
No. of turns in the middle coil	1152
No. of turns in the top and bottom coil	883
Average resistance of the middle coil	71.2 Ω
Average resistance of the top and bottom coil	42.3 Ω
Wire gauge	AWG 25
Wire material	Copper
Bobbin material	Aluminum
